# Genome-based analyses reveal a synonymy among *Halorubrum distributum* Zvyagintseva and Tarasov 1989; Oren and Ventosa 1996, *Halorubrum terrestre* Ventosa *et al*. 2004, *Halorubrum arcis* Xu *et al*. 2007 and *Halorubrum litoreum* Cui *et al*. 2007. Emended description of *Halorubrum distributum* Zvyagintseva and Tarasov 1989; Oren and Ventosa 1996

**DOI:** 10.1099/ijsem.0.003956

**Published:** 2020-01-23

**Authors:** Carmen Infante-Domínguez, Rafael R. de la Haba, Paulina Corral, Cristina Sanchez-Porro, David R. Arahal, Antonio Ventosa

**Affiliations:** ^1^​ Department of Microbiology and Parasitology, Faculty of Pharmacy, University of Sevilla, 41012 Sevilla, Spain; ^2^​ Department of Microbiology and Ecology, and Spanish Type Culture Collection (CECT), University of Valencia, 46980 Paterna (Valencia), Spain

**Keywords:** *Halorubrum*, taxonomy, synonym, emended description, *Halorubrum distributum*

## Abstract

A comparative taxonomic study of *Halorubrum distributum *, *Halorubrum terrestre *, *Halorubrum arcis* and *Halorubrum litoreum* was carried out using different approaches, 16S rRNA gene sequence analysis, multilocus sequence analysis (MLSA), phylogenomic analysis based on the comparison of the core genome, orthologous average nucleotide identity (OrthoANI), Genome-to-Genome Distance Calculator (GGDC), synteny plots and polar lipid profile (PLP). The MLSA study, using the five concatenated housekeeping genes *atpB*, *EF-2*, *glnA*, *ppsA* and *rpoB*′, and the phylogenomic analysis based on 1347 core translated gene sequences obtained from their genomes showed that *Halorubrum distributum* JCM 9100^T^, *Halorubrum terrestre* JCM 10247^T^, *Halorubrum arcis* JCM 13916^T^ and *Halorubrum litoreum* JCM 13561^T^ formed a robust cluster, clearly separated from the rest of species of the genus *Halorubrum *. The OrthoANI and digital DDH values, calculated by the GGDC, showed percentages among *Hrr. distributum* JCM 9100^T^, *Hrr. terrestre* JCM 10247^T^, *Hrr. arcis* JCM 13916^T^ and *Hrr. litoreum* JCM 13561^T^ that ranged from 98.1 to 97.5 %, and 84.0 to 78.0 %, respectively, while these values among those strains and the type strains of their most related species of *Halorubrum* were equal or lower than 90.8 and 41.2 %, respectively. Moreover, degree of synteny across the four genomes was very high, especially between the genomes of *Halorubrum litoreum* JCM 13561^T^ and *Halorubrum arcis* JCM 13916^T^. In addition, the PLP is quite similar among the four strains studied, showing a common pattern typical of the neutrophilic species of the genus *Halorubrum *. Overall, these data show that *Hrr. distributum*, *Hrr. terrestre*, *Hrr. arcis* and *Hrr. litoreum* constitute a single species. Thus, the latter three should be considered as later, heterotypic synonyms of *Hrr. distributum* based on the rules for priority of names. We propose an emended description of *Hrr. distributum*, including the features of *Hrr. terrestre*, *Hrr. arcis* and *Hrr. litoreum*.

The genus *Halorubrum* was proposed in 1995 by McGenity and Grant [1] in order to reclassify several species previously included in the genus *Halobacterium *. Two additional *Halorubrum* species, reclassified as members of the genus *Halorubrobacterium* [2], were lately transferred into this genus [3]. This genus was emended in 2009 by Oren *et al.* [4]. Currently, the genus *Halorubrum* is classified within the family *Halorubraceae *, order *Haloferacales *, class *Halobacteria* [5, 6], with *Halorubrum saccharovorum* as the type species. As of June 2019, the genus *Halorubrum *, with 39 species with validly published names, includes the largest number of species among all the genera within the haloarchaea [7, 8]. These species were isolated from diverse hypersaline habitats, such as saline and soda lakes, salterns, coastal sabkhas or saline soils, as well as from rock salt and salted foods [4, 8]. The genus *Halorubrum* includes rods or pleomorphic cells, Gram-variable, most species are motile and their colonies are red- to orange-pigmented. They are oxidase- and catalase-positive, extremely halophilic archaea, neutrophilic and in some cases alkaliphilic (optimum pH 9–10), such as *Halorubrum alkaliphilum *, *Halorubrum gandharaense *, *Halorubrum luteum *, *Halorubrum tibetense* and *Halorubrum vacuolatum *. Their major polar lipids are phosphatidylglycerol, phosphatidylglycerol phosphate methyl ester, phosphatidylglycerol sulfate and a sulfated mannosyl glucosyl diether. However, alkaliphilic species lack phosphatidylglycerol sulfate and glycolipids. Their genomic DNA G+C content range is 60.2–71.2 mol% [7, 8]. In the past, there has been a tendency to characterize and delineate new species of haloarchaea on the basis of their 16S rRNA gene sequence comparison, and at a later stage DNA–DNA hybridization (DDH) and phenotypic features, including the polar lipid analysis with their 16S rRNA-phylogenetically most closely related species. However, several studies pointed out that the use of the 16S rRNA gene was not adequate as a phylogenetic marker for this archaeal group due to the extensive horizontal gene transfer events, the presence of several rRNA operons (in some cases divergent) in their genomes and the low evolutionary rate of this gene [9–12]. Alternative methodologies such as the use of the *rpoB*′ gene or a multilocus sequence analysis (MLSA) based on the comparison of several single-copy housekeeping genes have been used [13, 14]. Recently, we have proposed the use of the five housekeeping genes *atpB*, *EF-2*, *glnA*, *ppsA* and *rpoB*′ for such phylogenetic analyses as an alternative to the 16S rRNA gene [15]. In this study, we determined that some well-stablished species of *Halorubrum* were not separate species, such as in the case of *Halorubrum ezzemoulense* and *Halorubrum chaoviator* [15, 16] and other similar situations might happen for other species of *Halorubrum *.

Here, we have studied in detail four species of *Halorubrum, Hrr. distributum* and *Hrr. terrestre*, isolated from saline soils [17, 18], *Hrr. arcis*, isolated from a saline lake [19] and *Hrr. litoreum*, isolated from a saltern [20], for which the draft genome sequences are available. The results show that they are members of the same species, *Hrr. distributum* being the species name with priority according to the International Code of Nomenclature of Prokaryotes [21], and thus it should be considered as an earlier synonym of the other three species of *Halorubrum *.

The 16S rRNA gene and MLSA phylogenetic analyses were carried out as described previously [14, 15]. Since some members of the class *Halobacteria* possess highly divergent 16S rRNA genes, we compared the reference 16S rRNA gene sequence (see in Table S1, available in the online verion of this article) of each of the analysed *Halorubrum* species with the 16S rRNA gene sequences extracted from the corresponding genome (partial 16S rRNA gene sequences <500 bp or low quality sequences were excluded). All the 16S rRNA gene sequences obtained from the genomes showed a similarity between 98.4–100 % with the 16S rRNA gene reference sequence. A more detailed analysis of the pairwise alignments unveiled that lower similarities (i.e. 98.4 %) were due to genome misassembly and, therefore, only microheterogeneities among 16S rRNA gene copies but not highly divergent copies were detected in the species of the genus *Halorubrum *. The nucleotide sequence of the 16S rRNA gene from the strains was aligned with arb 6.0.5 software package [22]. The sequence similarity analysis was carried out by comparing the 16S rRNA gene sequence of *Halorubrum distributum* JCM 9100^T^, *Halorubrum terrestre* VKM B-1739^T^, *Halorubrum arcis* AJ201^T^ and *Halorubrum litoreum* Fa-1^T^ with the known sequences of the *Halorubrum* species showed in Table S1, using the EzBioCloud tool (www.ezbiocloud.net/eztaxon; [23]) and arb 6.0.5 software. The analysis based on the complete 16S rRNA gene sequences showed the percentages of similarity included in Table S2. The 16S rRNA gene sequence of the type strain of *Hrr. distributum* JCM 9100^T^ with respect to those of *Hrr. terrestre* VKM B-1739^T^, *Hrr. arcis* AJ201^T^ and *Hrr. litoreum* Fa-1^T^ showed a similarity of 97.3, 95.8 and 98.6 %, respectively, which might be interpreted as those three strains belonging to different species than *Hrr. distributum*, according to the current accepted 16S rRNA gene sequence similarity threshold for species differentiation (98.65 % as a general limit, 98.2 % specifically for the phylum *Euryarchaeota *) [24, 25]. However, as indicated above, the 16S rRNA gene is not considered a reliable phylogenetic marker for this archaeal group and, therefore, these results must be regarded with caution and carefully checked. The phylogenetic study based on the 16S rRNA gene sequence comparison was performed by reconstructing phylogenetic trees using the following algorithms: neighbour-joining, maximum-parsimony and maximum-likelihood. Neighbour-joining distances were corrected according to the Jukes–Cantor matrix [26] and phylogenetic neighbour-joining and maximum parsimony trees were calculated with the arb program package version 6.0.5 [22]. Maximum-likelihood analysis was performed with PhyML version 3.3.20180214 [27] using the General Time Reversible (GTR+I+G) model of nucleotide substitution [28]. Base frequency per alignment position and positional variability by parsimony analysis of archaea taxa filters were applied in the sequence comparison and phylogenetic reconstruction analyses and the effects on the results were evaluated. To evaluate the robustness of the phylogenetic tree, a bootstrap analysis (1000 replications) was performed [29]. The phylogenetic tree based on the 16S rRNA gene reconstructed by neighbour-joining showed that *Hrr. distributum* JCM 9100^T^, *Hrr. terrestre* VKM B-1739^T^, *Hrr. arcis* AJ201^T^ and *Hrr. litoreum* Fa-1^T^ were phylogenetically related but they did not constitute a single cluster (Fig. 1). The topologies of phylogenetic trees inferred using the maximum-likelihood (Fig. S1) and maximum-parsimony algorithms were very similar to that of the tree reconstructed by neighbour-joining. As previously indicated, the comparison of the 16S rRNA gene sequences do not permit to determine in depth the phylogenetic relationships within the genus *Halorubrum* and thus an MLSA approach based on the comparison of partial sequences of the *atpB*, *EF-2*, *glnA*, *ppsA* and *rpoB*′ housekeeping genes (Table S1) has been recently recommended for this genus [15]. Fig. 2 shows the phylogenetic tree obtained by the concatenation of these five housekeeping genes, reconstructed by the maximum-likelihood algorithm following the same methodology as aforementioned. This tree shows a better phylogenetic separation of the species of *Halorubrum* and on the other hand, in this case *Hrr. distributum* JCM 9100^T^, *Hrr. terrestre* JCM 10247^T^, *Hrr. arcis* JCM 13916^T^ and *Hrr. litoreum* JCM 13561^T^ constituted a well-supported cluster, separated from other species of the genus *Halorubrum *. The percentage of similarity of the five concatenated genes of *Hrr. distributum* JCM 9100^T^, *Hrr. terrestre* JCM 10247^T^, *Hrr. arcis* JCM 13916^T^ and *Hrr. litoreum* JCM 13561^T^ ranged between 98.3–99.3 % and those of these four species with respect to the other type strains of *Halorubrum* were always below 95.6 % (Table S3), below the 96 % cut-off value established for species delineation within the genus *Halorubrum* [15].

**Fig. 1.**
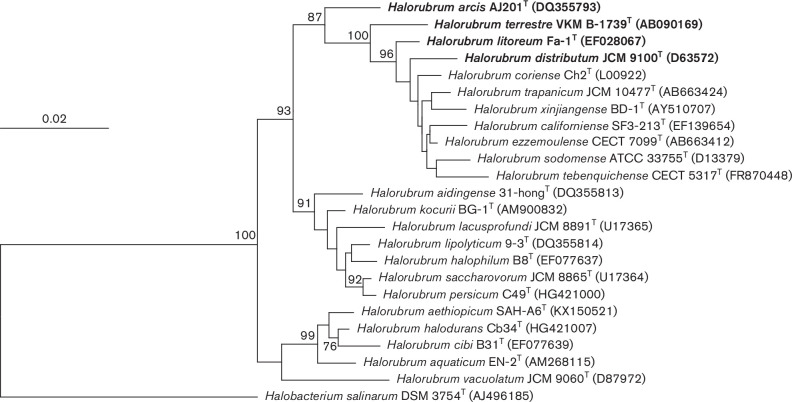
Neighbour-joining phylogenetic tree based on 16S rRNA gene sequences comparison showing the relationships between *Halorubrum distributum *, *Halorubrum terrestre *, *Halorubrum arcis* and *Halorrubrum litoreum* and other related species of the genus *Halorubrum *. The accession numbers of the sequences used are shown in parentheses after the strain designation. Bootstrap values (%) based on 1000 replicates are shown for branches with more than 70 % bootstrap support. *Halobacterium salinarum* DSM 3754^T^ was used as an outgroup. Bar, 0.02 substitutions per nucleotide position.

**Fig. 2.**
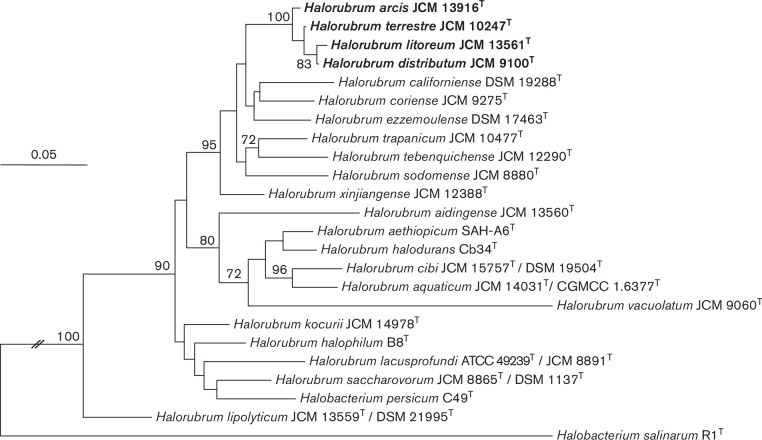
Maximum-likelihood phylogenetic tree based on the five housekeeping gene (*atpB*, *EF-2*, *glnA*, *ppsA* and *rpoB*′) concatenated sequences showing the relationship between *Halorubrum distributum *, *Halorubrum terrestre *, *Halorubrum arcis* and *Halorrubrum litoreum* and other related species of the genus *Halorubrum *. The accession numbers of the sequences used are shown in Table S1. Bootstrap values (%) based on 1000 replicates are indicated for branches above 70 %. *Halobacterium salinarum* DSM 3754^T^ was used as an outgroup. Bar, 0.05 substitutions per nucleotide position.

To increase the resolution, we carried out a phylogenomic analysis based on the 1347 core translated gene sequences obtained from the available genomes of *Hrr. distributum* JCM 9100^T^, *Hrr. terrestre* JCM 10247^T^, *Hrr. arcis* JCM 13916^T^ and *Hrr. litoreum* JCM 13561^T^ and the type strains of other related *Halorubrum* species (Tables S1 and S4). In brief, the translated gene sequences of all the predicted coding sequences of the genomes under study were compared by blast search in an all-versus-all mode to identify clusters of orthologous genes (OGs), that is, genes with a common ancestor present in two or more species. Those OGs shared among all taxa constituted the core genome of the studied genomes, formed by 1415 genes. From those, only the ones present in single copy per genome (a total of 1347 genes) were selected to reconstruct the phylogenomic tree. Enveomics collection toolbox [30] was used to carry out the aforementioned OG identification and core-genome selection. The 1347 translated gene sequences for each species were aligned with muscle version 3.8.31 [31] and subsequently concatenated. An approximate maximum-likelihood tree was reconstructed using FastTree version 2.1.9 [32] with the JTT replacement matrix [33] under the CAT approximation (single rate for each site) with 20 rate categories. Local support values were estimated with the Shimodaira–Hasegawa test [34]. As shown in Fig. 3, the overall topology of the phylogenetic tree was in agreement with the MLSA tree. *Hrr. distributum* JCM 9100^T^, *Hrr. terrestre* JCM 10247^T^, *Hrr. arcis* JCM 13916^T^ and *Hrr. litoreum* JCM 13561^T^ formed a maximally supported subtree, clearly separated from the rest of species of *Halorubrum* (Fig. 3). A total of 2571 common OGs are shared among the four strains, while pairwise comparisons between the four species studied yielded 2665–2769 shared OGs (Fig. 4).

**Fig. 3.**
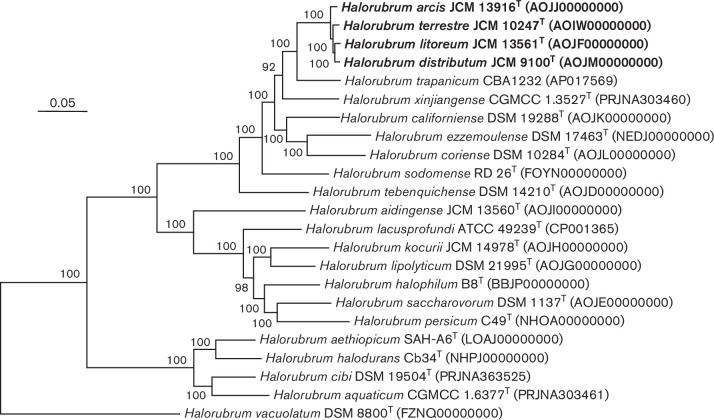
Approximately maximum-likelihood core protein phylogenomic tree including the genomes of *Halorubrum distributum *, *Halorubrum terrestre *, *Halorubrum arcis* and *Halorrubrum litoreum* and other related species of the genus *Halorubrum *. This tree was based on the JTT substitution matrix under the CAT approximation with 20 rate categories from the alignment of 1342 shared orthologous single-copy translated genes of these genomes. All genomes were retrieved from GenBank (Table S1). Local support values based on the Shimodaira–Hasegawa test over 70 % are shown above the branch. Bar, 0.05 substitutions per nucleotide position.

**Fig. 4.**
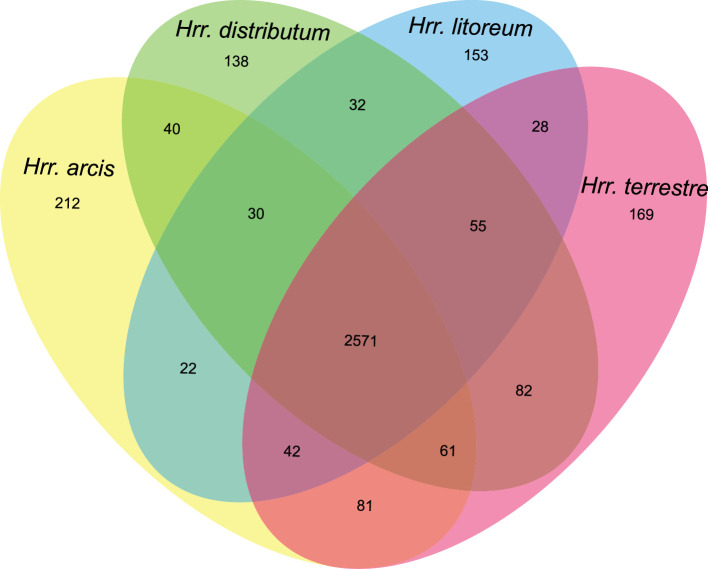
Venn diagram showing the core orthologous and unique proteins for the type strains of *Halorubrum distributum* JCM 9100^T^, *Halorubrum terrestre* JCM 10247^T^, *Halorubrum arcis* JCM 13916^T^ and *Halorubrum litoreum* JCM 13561^T^.

The OrthoANI percentages, determined on the basis of usearch comparison of the genome sequences of *Hrr. distributum* JCM 9100^T^, *Hrr. terrestre* JCM 10247^T^, *Hrr. arcis* JCM 13916^T^ and *Hrr. litoreum* JCM 13561^T^ with respect to the type strains of the related *Halorubrum* species, as implemented in OrthoANIu tool [35], indicate that the cluster formed by these strains showed a range of 97.5–98.1 % among these strains, while the percentage with respect to the related species of *Halorubrum* was equal or lower than 90.8 % (Table 1). The threshold of 95–96 % defined for species delineation [36–38] clearly supports the placement of these four strains within a single species.

**Table 1.** GGDC (lower triangle) and OrthoANIu (upper triangle) values for the type strains of the species of the genus *Halorubrum* with available genomes Cells are filled with darker colours for higher values.

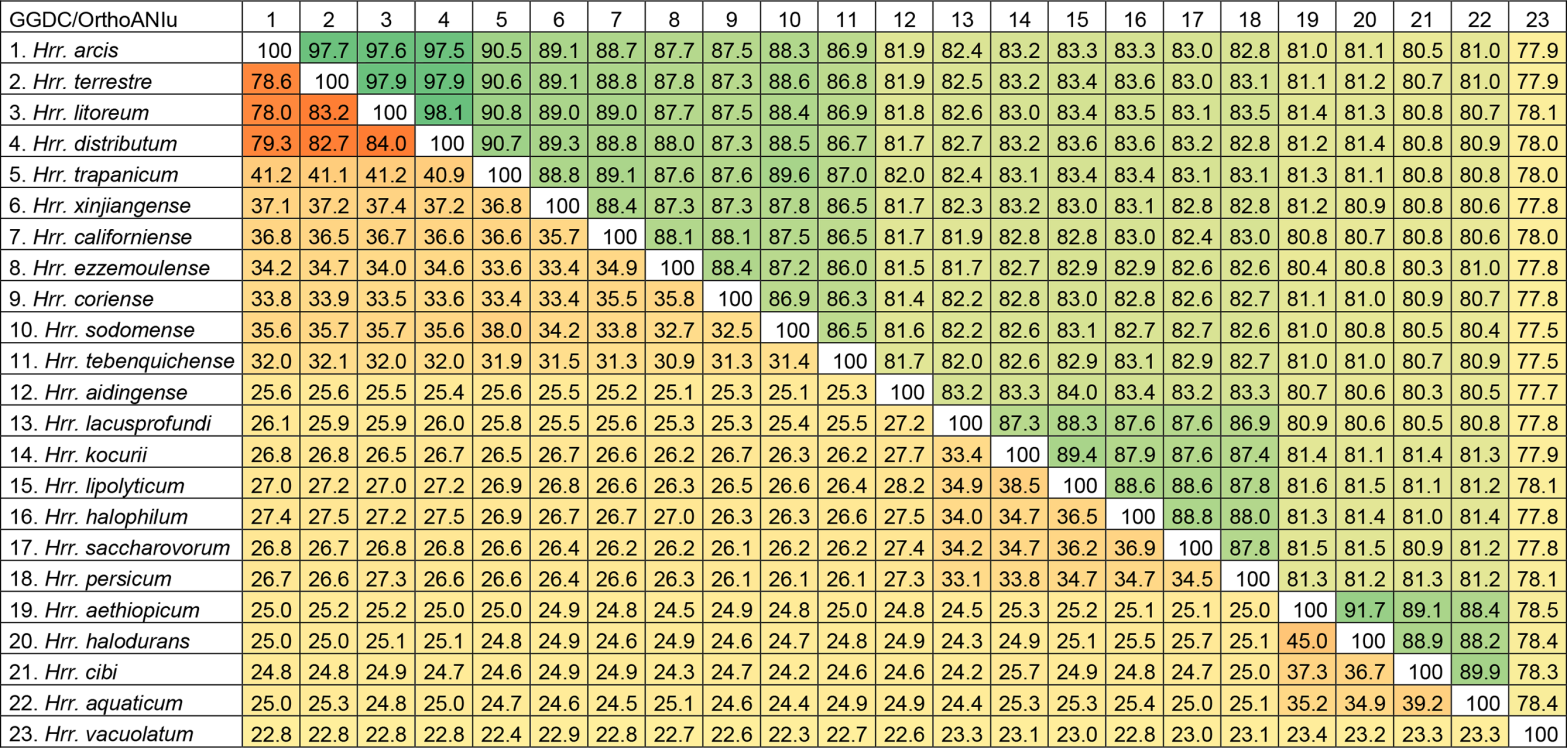

We also calculated the digital DDH values, determined online (http://ggdc.dsmz.de/distcalc2.php) using the Genome-to-Genome Distance Calculator (GGDC) version 2.0 as described by Meier-Kolthoff *et al.* [39]. The estimated digital DDH values were calculated using formula two at the GGDC website, originally described by Auch *et al.* [40] and updated by Meier-Kolthoff *et al.* [39]. The digital DDH values among *Hrr. distributum* JCM 9100^T^, *Hrr. terrestre* JCM 10247^T^, *Hrr. arcis* JCM 13916^T^ and *Hrr. litoreum* JCM 13561^T^ ranged from 84.0 to 78.0 %, but the values among these strains and the type strains of species of *Halorubrum* was equal or lower than 41.2 % (Table 1), showing unequivocally that the four species under study constitute a single species of *Halorubrum *, clearly separated from the rest of species of this genus. The cut-off for this parameter is well stablished as 70 % for species delineation [38, 39].

Given the high values of orthoANI and digital DDH among the four species *Hrr. distributum* JCM 9100^T^, *Hrr. terrestre* JCM 10247^T^, *Hrr. arcis* JCM 13916^T^ and *Hrr. litoreum* JCM 13561^T^, we inspected the conservation of blocks of order across those genomes by means of synteny plots constructed using the edgar 2.0 software platform [41]. As shown in Fig. S2, gene co-localization is highly conserved in those four genomes, especially remarkable between *Hrr. litoreum* JCM 13561^T^ and *Hrr. arcis* JCM 13916^T^ genomes, also supporting the existence of a synonymy among the four species.

DNA G+C content was calculated from the genomic sequences (Table S1) and compared to the DNA G+C content provided in the species descriptions. For *Hrr. distributum* JCM 9100^T^, *Hrr. terrestre* JCM 10247^T^, *Hrr. arcis* JCM 13916^T^ and *Hrr. litoreum* JCM 13561^T^ the *in silico* values were 68.1, 68.0, 67.3 and 68.9 mol%, respectively, whereas the wet values were 63.9 (T*_m_*) [17], 64.4 (T*_m_*) [18], 65.7 (T*_m_*) [19] and 64.9 mol% (T*_m_*) [20]. Therefore, significant discrepancies exist between *in silico* and conventional G+C values probably due to the inaccuracies of the applied conventional method and, accordingly, emendation of the species descriptions seems necessary. Although *in silico* G+C values among the strains belonging to the same species should not vary more than 1 % [42], in this case, all the strains are within this 1 % difference with respect to *Hrr. distributum* JCM 9100^T^, the type strain of the species name with priority.

For the polar lipid profile (PLP) analyses, the cell biomass of the strains was obtained after 10 days of aerobic incubation in modified SW20 liquid medium under optimal conditions: 20 % (w/v) NaCl, 37 °C and pH 7.5. The extraction of membrane polar lipids of halophilic archaea and their profile detection were performed following the methods described by Corral *et al.* [43, 44]. The lipids pattern was obtained by one-dimensional high-performance thin-layer chromatography (HPTLC) and then revealed by universal and specific stains for the identification of chemical nature of the polar lipid bands present in the HPTLC plate [43–46]. The lipid extracts of *Halobacterium salinarum* DSM 3754^T^, *Halorubrum saccharovorum* DSM 1137^T^ and *Halorubrum tibetense* JCM 11889^T^, as representative of the alkaliphilic species, were used as reference in this study.

The thin-layer chromatography results of the polar lipids (Fig. S3) revealed that *Hrr. distributum* JCM 9100^T^, *Hrr. terrestre* JCM 10247^T^, *Hrr. arcis* JCM 13916^T^ and *Hrr. litoreum* JCM 13561^T^ possess a similar pattern of polar lipid profile, showing in common the major lipids: phosphatidylglycerol, phosphatidylglycerol phosphate methyl ester, phosphatidylglycerol sulfate and the main polar lipid component of this genus: sulfated mannosyl glucosyl diether. Biphosphatidylglycerol was also found and minor phospholipids were also detected. The polar lipid profile of all these strains possessed all major lipids described for neutrophilic species of the genus *Halorubrum* [1, 4].

In summary, the genome-based study shows that *Hrr. distributum* JCM 9100^T^, *Hrr. terrestre* JCM 10247^T^, *Hrr. arcis* JCM 13916^T^ and *Hrr. litoreum* JCM 13561^T^ constitute a single species. In accordance to the International Code of Nomenclature of Prokaryotes [21], the name *Hrr. distributum* has priority and thus, *Hrr. terrestre*, *Hrr. arcis* and *Hrr. litoreum* should be considered as later heterotypic synonyms of *Hrr. distributum*. Based on these corroborations we propose the emended description of the species *Hrr. distributum*, including the features of *Hrr. terrestre*, *Hrr. arcis* and *Hrr. litoreum.*

## Emended description of *Halorubrum distributum* Zvyagintseva and Tarasov 1989; Oren and Ventosa 1996

*Halorubrum distributum* (dis.tri.bu′tum. L. neut. part. adj. *distributum*, distributed [widely]).

The description is that of Zvyagintseva and Tarasov [17] and Oren and Ventosa [3] with the following modifications: cells are rod-shaped, pleomorphic, flat or disk-shaped, aerobic growth occurs at 12–30 % (w/v) NaCl, pH 5.0–9.0 and 20–55 °C. Optimum NaCl concentration, pH and temperature for growth are 20–25 % (w/v), pH 7.0–7.5 and 37–42 °C. The polar lipid profile includes phosphatidylglycerol, phosphatidylglycerol phosphate methyl ester, phosphatidylglycerol sulfate and one glycolipid chromatographically identical to sulfated mannosyl glycosyl diether. Biphosphatidylglycerol is also found as a minor component, and minor phospholipids are also detected.

The G+C content of the genomic DNA is 67.3–68.9 mol% (genome).

The type strain, 1m^T^ (=VKM B-1733^T^=ATCC 51197^T^=CGMCC 1.3491^T^=CIP 105238^T^=JCM 9100^T^=NCIMB 13203^T^), was isolated from a saline soil sample. The DNA G+C content of this strain is 68.1 mol% (genome).

*Hrr. terrestre* 4p^T^ (=VKM B-1739^T^=CIP 108318^T^=DSM 23453^T^=JCM 10247^T^), *Hrr. arcis* AJ201^T^ (=CGMCC 1.5343^T^=DSM 23454^T^=JCM 13916^T^) and *Hrr. litoreum* Fa-1^T^ (=CGMCC 1.5336^T^=DSM 23497^T^=JCM 13561^T^) are additional strains of *Halorubrum distributum *.

## Supplementary Data

Supplementary material 1
