# Supplementary material for: Genome-based analyses reveal a synonymy among Halorubrum distributum Zvyagintseva and Tarasov 1989; Oren and Ventosa 1996, Halorubrum terrestre Ventosa et al. 2004, Halorubrum arcis Xu et al. 2007 and Halorubrum litoreum Cui et al. 2007. Emended description of Halorubrum distributum Zvyagintseva and Tarasov 1989; Oren and Ventosa 1996
Source: Int J Syst Evol Microbiol. 2020 Jan 23;70(3):1698–705. doi: 10.1099/ijsem.0.003956 (PMC7386789; doi:10.1099/ijsem.0.003956)
Supplement: Supplementary material 1 [file ijsem-70-1698-s001.pdf]

**International Journal of Systematic and Evolutionary Microbiology**

**Supplementary Material**

# Genome-based analyses reveal a synonymy among *Halorubrum distributum* Zvyagintseva and Tarasov 1989; Oren and Ventosa 1996, *Halorubrum terrestre* Ventosa *et al.* 2004, *Halorubrum arcis* Xu *et al.* 2007 and *Halorubrum litoreum* Cui *et al.* 2007. Emended description of *Halorubrum distributum* Zvyagintseva and Tarasov 1989; Oren and Ventosa 1996

Carmen Infante-Domínguez[1]†, Rafael R. de la Haba[1]†, Paulina Corral[1], Cristina Sánchez-Porro[1], David R. Arahal[2] and Antonio Ventosa[1*]

[1]Department of Microbiology and Parasitology, Faculty of Pharmacy, University of Sevilla, 41012 Sevilla, Spain

[2]Department of Microbiology and Ecology, and Spanish Type Culture Collection (CECT), University of Valencia, 46980 Paterna (Valencia), Spain

[*]Corresponding author (ventosa@us.es)

†These authors contributed equally to this work.

**Supplementary Table S1.** Type strains of species of the genus *Halorubrum* with available genome used in this study, showing the accession numbers for the sequences deposited in GenBank/EMBL/DDBJ and the DNA G+C content, calculated on the basis of the genome sequence. ND, not determined.

| **Strain** | **16S rRNA** | ***atpB*** | ***EF-2*** | ***glnA*** | ***ppsA*** | ***rpoB'*** | **Genome** | **DNA G+C (%, genome)** |
|---|---|---|---|---|---|---|---|---|
| *Halorubrum aethiopicum* SAH-A6$^T$ | KX150521 | LOAJ00000000 | LOAJ00000000 | LOAJ00000000 | LOAJ00000000 | MF039476 | LOAJ00000000 | 68.0 |
| *Halorubrum aidingense* 31-hong$^T$†/JCM 13560$^T$‡ | DQ355813† | HQ149374‡ | HQ149406‡ | KM107963‡ | AOJI00000000‡ | AB477176‡ | AOJI00000000‡ | 67.2 |
| *Halorubrum aquaticum* EN-2$^T$†/CGMCC 1.6377$^T$‡/JCM 14031$^T$§ | AM268115† | PRJNA303461‡ | KM980074‡ | PRJNA303461‡ | PRJNA303461‡ | AB820296§ | FOPZ00000000‡ | 67.4 |
| *Halorubrum arcis* AJ201$^T$†/JCM 13916$^T$‡ | DQ355793† | KM105848‡ | KM107920‡ | KM107964‡ | KM107932‡ | AB477178‡ | AOJJ00000000‡ | 67.3 |
| *Halorubrum californiense* SF3-213$^T$†/DSM 19288$^T$‡ | EF139654† | ND | AOJK00000000‡ | AOJK00000000‡ | AOJK00000000‡ | AOJK00000000‡ | AOJK00000000‡ | 66.2 |
| *Halorubrum cibi* B31$^T$†/ DSM 19504$^T$‡/JCM 15757 $^T$§ | EF077639† | PRJNA363525‡ | PRJNA363525‡ | PRJNA363525‡ | PRJNA363525‡ | AB820297§ | FXTD00000000‡ | 66.8 |
| *Halorubrum coriense* Ch2$^T$†/JCM 9275$^T$‡/ DSM 10284$^T$§ | L00922† | HQ149357‡ | HQ149407‡ | KM107966‡ | KM107934‡ | HQ149490‡ | AOJL00000000§ | 67.0 |
| *Halorubrum distributum* JCM 9100$^T$ | D63572 | HQ149358 | HQ149408 | KM107967 | KM107935 | HQ149491 | AOJM00000000 | 68.1 |
| *Halorubrum ezzemoulense* CECT 7099$^T$†/DSM 17463$^T$‡ | AB663412† | NEDJ00000000‡ | NEDJ00000000‡ | NEDJ00000000‡ | NEDJ00000000‡ | NEDJ00000000‡ | NEDJ00000000‡ | 66.6 |
| *Halorubrum halodurans* Cb34$^T$ | HG421007 | KJ152237 | KJ152279 | KJ152363 | KJ152413 | KJ152360 | NHPJ00000000 | 67.6 |
| *Halorubrum halophilum* B8$^T$ | EF077637 | BBJP00000000 | BBJP00000000 | BBJP00000000 | BBJP00000000 | BBJP00000000 | BBJP00000000 | 65.1 |
| *Halorubrum kocurii* BG-1$^T$†/JCM 14978$^T$‡ | AM900832† | AOJH00000000‡ | AOJH00000000‡ | AOJH00000000‡ | AOJH00000000‡ | AOJH00000000‡ | AOJH00000000‡ | 66.9 |
| *Halorubrum lacusprofundi* JCM 8891$^T$†/ATCC 49239$^T$‡ | U17365† | HQ149375‡ | CP001365‡ | CP001365‡ | CP001365‡ | AB477181† | CP001365‡ | 64.0 |
| *Halorubrum lipolyticum* 9-3$^T$†/JCM 13559$^T$‡/DSM 21995$^T$§ | DQ355814† | KM105851‡ | AOJG00000000§ | KM107969‡ | KM107937‡ | AB477182‡ | AOJG00000000§ | 68.0 |
| *Halorubrum litoreum* Fa-1$^T$†/JCM 13561$^T$‡ | EF028067† | KM105852‡ | KM107923‡ | KM107970‡ | KM107938‡ | AB477183‡ | AOJF00000000‡ | 68.9 |
| *Halorubrum persicum* C49$^T$ | HG421000 | KJ152247 | KJ152276 | KJ152362 | KM107947 | KJ152361 | NHOA00000000 | 65.9 |
| *Halorubrum saccharovorum* JCM 8865$^T$†/DSM 1137$^T$‡ | U17364† | HQ149376† | HQ149409† | KM107971† | AOJE00000000‡ | AB477184† | AOJE00000000‡ | 66.9 |

| | | | | | | | | |
|---|---|---|---|---|---|---|---|---|
| *Halorubrum sodomense* ATCC 33755$^T$†/JCM 8880$^T$‡/RD 26$^T$§ | D13379† | HQ149377‡ | HQ149410‡ | KM107972‡ | KM107939‡ | AB477185‡ | FOYN00000000§ | 69.0 |
| *Halorubrum tebenquichense* CECT 5317$^T$†/JCM 12290$^T$‡/DSM 14210$^T$§ | FR870448† | KM105853‡ | KM107924‡ | KM107973‡ | KM107940‡ | AB477186‡ | AOJD00000000§ | 68.0 |
| *Halorubrum terrestre* VKM B-1739$^T$†/JCM 10247$^T$‡ | AB090169† | KM105854‡ | KM107925‡ | KM107974‡ | KM107941‡ | AB477187‡ | AOIW00000000‡ | 68.0 |
| *Halorubrum trapanicum* JCM 10477$^T$†/CBA1232‡ | AB663424† | KM105856† | KM107927† | KM107976† | KM107943† | AB477189† | AP017569‡ | 69.6 |
| *Halorubrum vacuolatum* JCM 9060$^T$†/DSM 8800$^T$‡ | D87972† | HQ149378† | HQ149411† | KM107977† | KM107944† | AB477190† | FZNQ00000000‡ | 62.9 |
| *Halorubrum xinjiangense* BD-1$^T$†/JCM 12388$^T$‡/ CGMCC 1.3527$^T$§ | AY510707† | KM105857‡ | KM107928‡ | KM107978‡ | KM107945‡ | AB477191‡ | FNBO00000000§ | 68.3 |

The strain designation corresponding to each sequence accession number is indicated with the symbol †, ‡ or §.

**Supplementary Table S2.** Similarity matrix based on 16S rRNA gene sequence comparison among the analyzed type strains of species of the genus *Halorubrum*. Cells are filled with darker colors for higher values.

| | 1 | 2 | 3 | 4 | 5 | 6 | 7 | 8 | 9 | 10 | 11 | 12 | 13 | 14 | 15 | 16 | 17 | 18 | 19 | 20 | 21 | 22 | 23 |
|---|---|---|---|---|---|---|---|---|---|---|---|---|---|---|---|---|---|---|---|---|---|---|---|
| 1. *Hrr arcis* | 100 | | | | | | | | | | | | | | | | | | | | | | |
| 2. *Hrr terrestre* | 96.9 | 100 | | | | | | | | | | | | | | | | | | | | | |
| 3. *Hrr. litoreum* | 97.0 | 97.9 | 100 | | | | | | | | | | | | | | | | | | | | |
| 4. *Hrr. distributum* | 95.8 | 97.3 | 98.6 | 100 | | | | | | | | | | | | | | | | | | | |
| 5. *Hrr. trapanicum* | 96.5 | 97.3 | 98.4 | 98.0 | 100 | | | | | | | | | | | | | | | | | | |
| 6. *Hrr. xinjiangense* | 96.6 | 97.3 | 98.4 | 98.0 | 98.8 | 100 | | | | | | | | | | | | | | | | | |
| 7. *Hrr. californiense* | 95.7 | 96.8 | 97.9 | 97.6 | 98.7 | 98.1 | 100 | | | | | | | | | | | | | | | | |
| 8. *Hrr. ezzemoulense* | 96.5 | 97.6 | 98.8 | 98.5 | 99.2 | 98.5 | 98.9 | 100 | | | | | | | | | | | | | | | |
| 9. *Hrr. coriense* | 96.3 | 97.3 | 98.8 | 98.5 | 98.5 | 98.6 | 98.4 | 99.2 | 100 | | | | | | | | | | | | | | |
| 10. *Hrr. sodomense* | 96.3 | 97.3 | 97.9 | 98.0 | 98.5 | 98.1 | 98.4 | 98.8 | 98.4 | 100 | | | | | | | | | | | | | |
| 11. *Hrr. tebenquichense* | 96.4 | 96.8 | 97.6 | 97.3 | 98.2 | 97.7 | 97.9 | 98.5 | 97.9 | 98.2 | 100 | | | | | | | | | | | | |
| 12. *Hrr. aidingense* | 96.8 | 95.5 | 96.3 | 94.9 | 95.8 | 95.8 | 95.0 | 95.4 | 95.3 | 95.7 | 95.5 | 100 | | | | | | | | | | | |
| 13. *Hrr. lacusprofundi* | 97.1 | 96.0 | 95.7 | 94.6 | 95.0 | 95.0 | 94.1 | 95.0 | 94.8 | 95.2 | 95.5 | 98.1 | 100 | | | | | | | | | | |
| 14. *Hrr. kocurii* | 97.3 | 96.3 | 96.3 | 95.1 | 95.9 | 95.8 | 95.5 | 96.0 | 95.5 | 96.1 | 95.8 | 98.4 | 98.4 | 100 | | | | | | | | | |
| 15. *Hrr. lipolyticum* | 97.6 | 96.7 | 96.4 | 95.2 | 95.6 | 95.6 | 94.8 | 95.7 | 95.4 | 95.6 | 96.0 | 98.1 | 98.5 | 99.1 | 100 | | | | | | | | |
| 16. *Hrr. halophilum* | 98.0 | 96.3 | 96.1 | 94.8 | 95.4 | 95.3 | 94.7 | 95.5 | 95.1 | 95.4 | 95.8 | 97.8 | 98.3 | 98.7 | 99.0 | 100 | | | | | | | |
| 17. *Hrr. saccharovorum* | 98.2 | 96.4 | 96.2 | 95.0 | 95.7 | 95.5 | 95.0 | 95.8 | 95.2 | 95.6 | 96.3 | 97.5 | 98.3 | 98.7 | 98.9 | 99.0 | 100 | | | | | | |
| 18. *Hrr. persicum* | 97.7 | 96.1 | 95.9 | 94.7 | 95.5 | 95.2 | 94.9 | 95.6 | 94.9 | 95.4 | 96.3 | 97.1 | 98.0 | 98.5 | 98.7 | 98.7 | 99.5 | 100 | | | | | |
| 19. *Hrr. aethiopicum* | 96.0 | 94.6 | 94.8 | 93.6 | 94.9 | 94.4 | 94.5 | 94.4 | 93.9 | 94.6 | 94.6 | 96.0 | 96.3 | 97.1 | 97.1 | 97.0 | 96.8 | 96.9 | 100 | | | | |
| 20. *Hrr. halodurans* | 95.9 | 94.6 | 95.0 | 93.9 | 95.1 | 94.6 | 94.5 | 94.6 | 94.1 | 94.9 | 94.9 | 96.4 | 96.3 | 97.3 | 97.1 | 96.9 | 96.6 | 96.9 | 99.1 | 100 | | | |
| 21. *Hrr. cibi* | 95.6 | 93.9 | 94.3 | 93.2 | 94.4 | 94.0 | 93.9 | 93.9 | 93.4 | 94.1 | 94.4 | 95.7 | 95.6 | 96.6 | 96.4 | 96.5 | 96.3 | 96.3 | 98.4 | 98.9 | 100 | | |
| 22. *Hrr. aquaticum* | 96.0 | 94.1 | 94.4 | 93.3 | 94.7 | 94.3 | 94.3 | 94.2 | 93.7 | 94.4 | 94.5 | 95.9 | 96.0 | 96.8 | 96.8 | 97.0 | 96.8 | 96.8 | 98.6 | 98.5 | 98.0 | 100 | |
| 23. *Hrr. vacuolatum* | 94.5 | 94.1 | 93.9 | 93.0 | 94.0 | 94.3 | 93.6 | 93.8 | 93.4 | 93.8 | 93.8 | 95.0 | 95.0 | 95.5 | 95.8 | 95.5 | 95.7 | 95.6 | 96.3 | 96.0 | 96.1 | 96.1 | 100 |

**Supplementary Table S3.** Similarity matrix based on the MLSA concatenated gene sequence comparison among the analyzed type strains of species of the genus *Halorubrum*. Cells are filled with darker colors for higher values.

| | 1 | 2 | 3 | 4 | 5 | 6 | 7 | 8 | 9 | 10 | 11 | 12 | 13 | 14 | 15 | 16 | 17 | 18 | 19 | 20 | 21 | 22 | 23 |
|---|---|---|---|---|---|---|---|---|---|---|---|---|---|---|---|---|---|---|---|---|---|---|---|
| 1. *Hrr arcis* | 100 | | | | | | | | | | | | | | | | | | | | | | |
| 2. *Hrr terrestre* | 98.9 | 100 | | | | | | | | | | | | | | | | | | | | | |
| 3. *Hrr. litoreum* | 98.4 | 98.7 | 100 | | | | | | | | | | | | | | | | | | | | |
| 4. *Hrr. distributum* | 98.3 | 99.1 | 99.3 | 100 | | | | | | | | | | | | | | | | | | | |
| 5. *Hrr. trapanicum* | 95.3 | 95.1 | 95.0 | 95.1 | 100 | | | | | | | | | | | | | | | | | | |
| 6. *Hrr. xinjiangense* | 95.6 | 95.2 | 95.1 | 95.1 | 94.9 | 100 | | | | | | | | | | | | | | | | | |
| 7. *Hrr. californiense* | 94.7 | 94.3 | 94.1 | 94.1 | 93.7 | 94.3 | 100 | | | | | | | | | | | | | | | | |
| 8. *Hrr. ezzemoulense* | 95.2 | 95.0 | 95.1 | 95.1 | 94.4 | 94.6 | 94.4 | 100 | | | | | | | | | | | | | | | |
| 9. *Hrr. coriense* | 95.0 | 94.6 | 95.1 | 95.1 | 94.2 | 95.1 | 93.9 | 95.3 | 100 | | | | | | | | | | | | | | |
| 10. *Hrr. sodomense* | 94.1 | 94.1 | 94.3 | 94.2 | 94.5 | 94.3 | 92.9 | 93.5 | 93.7 | 100 | | | | | | | | | | | | | |
| 11. *Hrr. tebenquichense* | 94.0 | 93.9 | 94.2 | 94.2 | 94.9 | 94.2 | 93.7 | 94.4 | 94.3 | 93.5 | 100 | | | | | | | | | | | | |
| 12. *Hrr. aidingense* | 91.2 | 91.0 | 90.9 | 90.7 | 91.1 | 91.3 | 90.0 | 90.9 | 90.5 | 90.6 | 90.6 | 100 | | | | | | | | | | | |
| 13. *Hrr. lacusprofundi* | 91.9 | 91.9 | 91.6 | 91.8 | 92.1 | 92.6 | 91.0 | 91.6 | 91.5 | 92.1 | 91.2 | 90.9 | 100 | | | | | | | | | | |
| 14. *Hrr. kocurii* | 93.6 | 93.7 | 93.6 | 93.7 | 93.5 | 94.3 | 92.3 | 93.7 | 93.8 | 92.9 | 93.2 | 92.2 | 93.9 | 100 | | | | | | | | | |
| 15. *Hrr. lipolyticum* | 91.1 | 91.3 | 91.1 | 91.1 | 90.8 | 91.5 | 89.0 | 90.7 | 91.4 | 90.8 | 90.1 | 89.4 | 90.0 | 92.7 | 100 | | | | | | | | |
| 16. *Hrr. halophilum* | 94.0 | 94.1 | 93.5 | 93.6 | 94.1 | 94.0 | 92.1 | 93.3 | 93.3 | 93.5 | 93.3 | 92.0 | 93.8 | 95.4 | 91.9 | 100 | | | | | | | |
| 17. *Hrr. saccharovorum* | 93.5 | 93.3 | 93.7 | 93.7 | 93.8 | 93.4 | 91.6 | 93.0 | 93.2 | 93.0 | 92.5 | 91.5 | 93.5 | 94.9 | 91.9 | 95.1 | 100 | | | | | | |
| 18. *Hrr. persicum* | 93.0 | 93.0 | 92.9 | 93.0 | 93.3 | 93.1 | 92.1 | 93.2 | 92.3 | 92.8 | 92.6 | 91.7 | 92.9 | 94.6 | 90.9 | 93.9 | 94.5 | 100 | | | | | |
| 19. *Hrr. aethiopicum* | 92.6 | 92.7 | 92.7 | 92.7 | 92.3 | 92.4 | 91.0 | 92.5 | 91.9 | 92.1 | 92.7 | 91.6 | 91.5 | 93.8 | 90.5 | 92.6 | 92.7 | 92.9 | 100 | | | | |
| 20. *Hrr. halodurans* | 93.0 | 93.0 | 92.7 | 92.9 | 92.3 | 93.0 | 91.4 | 92.5 | 92.3 | 92.4 | 93.0 | 91.5 | 91.2 | 93.6 | 90.4 | 92.6 | 92.2 | 92.7 | 97.0 | 100 | | | |
| 21. *Hrr. cibi* | 91.8 | 91.8 | 91.8 | 91.9 | 91.4 | 91.8 | 90.6 | 91.8 | 91.3 | 91.6 | 91.9 | 91.4 | 90.7 | 92.6 | 89.8 | 91.6 | 91.5 | 91.5 | 94.9 | 94.9 | 100 | | |
| 22. *Hrr. aquaticum* | 92.0 | 91.9 | 91.9 | 92.0 | 91.8 | 92.3 | 91.3 | 91.8 | 91.6 | 91.5 | 92.0 | 91.8 | 91.1 | 92.9 | 90.1 | 92.0 | 91.6 | 91.9 | 95.0 | 95.0 | 95.8 | 100 | |
| 23. *Hrr. vacuolatum* | 88.1 | 88.0 | 87.9 | 87.9 | 88.6 | 88.6 | 86.7 | 88.2 | 88.3 | 87.7 | 88.5 | 87.0 | 87.9 | 89.0 | 87.0 | 88.4 | 88.3 | 88.1 | 89.5 | 89.2 | 88.4 | 89.2 | 100 |

**Supplementary Table S4.** Genomic traits of the genome sequences used in this study.

| Strain | GenBank/EMBL/ DDBJ accession no. | Length (bp) | # contigs | N50 | Completeness (%) | Contamination (%) | # genes | # CDS | # rRNA | # tRNA | DNA G+C (mol%) |
|---|---|---|---|---|---|---|---|---|---|---|---|
| *Halorubrum aethiopicum* SAH-A6$^T$ | LOAJ00000000 | 3,325,770 | 3 | 3,045,622 | 97.92 | 0 | 3,261 | 3,204 | 6 | 49 | 68.0 |
| *Halorubrum aidingense* JCM 13560$^T$ | AOJI00000000 | 3,108,525 | 37 | 208,041 | 98.16 | 0.95 | 3,021 | 2,961 | 5 | 53 | 67.2 |
| *Halorubrum aquaticum* CGMCC 1.6377$^T$ | FOPZ00000000 | 3,184,102 | 40 | 151,981 | 99.00 | 0 | 3,173 | 3,119 | 5 | 47 | 67.4 |
| *Halorubrum arcis* JCM 13916$^T$ | AOJJ00000000 | 3,382,601 | 110 | 85,701 | 99.49 | 0.76 | 3,356 | 3,289 | 15 | 50 | 67.3 |
| *Halorubrum californiense* DSM 19288$^T$ | AOJK00000000 | 3,682,872 | 83 | 100,676 | 97.07 | 0.76 | 3,583 | 3,532 | 3 | 46 | 66.2 |
| *Halorubrum cibi* DSM 19504$^T$ | FXTD00000000 | 3,176,020 | 35 | 243,642 | 98.92 | 0 | 3,146 | 3,094 | 3 | 47 | 66.8 |
| *Halorubrum coriense* DSM 10284$^T$ | AOJL00000000 | 3,645,313 | 69 | 81,344 | 98.73 | 0.76 | 3,583 | 3,526 | 4 | 51 | 67.0 |
| *Halorubrum distributum* JCM 9100$^T$ | AOJM00000000 | 3,307,369 | 83 | 71,130 | 99.49 | 0 | 3,231 | 3,172 | 10 | 47 | 68.1 |
| *Halorubrum ezzemoulense* DSM 17463$^T$ | NEDJ00000000 | 3,603,550 | 155 | 40,831 | 99.85 | 0.19 | 3,651 | 3,585 | 7 | 57 | 66.6 |
| *Halorubrum halodurans* Cb34$^T$ | NHPJ00000000 | 3,320,122 | 143 | 42,928 | 94.17 | 0 | 3,368 | 3,306 | 3 | 56 | 67.6 |
| *Halorubrum halophilum* B8$^T$ | BBJP00000000 | 3,677,984 | 103 | 98,410 | 98.04 | 0.38 | 3,595 | 3,533 | 4 | 56 | 65.1 |
| *Halorubrum kocurii* JCM 14978$^T$ | AOJH00000000 | 3,619,738 | 105 | 65,834 | 97.78 | 1.9 | 3,510 | 3,457 | 4 | 47 | 66.9 |
| *Halorubrum lacusprofundi* ATCC 49239$^T$ | CP001365 | 3,692,576 | 3 | 2,735,295 | 99.76 | 0 | 3,656 | 3,595 | 9 | 50 | 64.0 |
| *Halorubrum lipolyticum* DSM 21995$^T$ | AOJG00000000 | 3,425,042 | 41 | 148,576 | 99.30 | 0.38 | 3,282 | 3,229 | 4 | 47 | 68.0 |
| *Halorubrum litoreum* JCM 13561$^T$ | AOJF00000000 | 3,137,757 | 63 | 111,876 | 99.49 | 0 | 3,082 | 3,025 | 7 | 48 | 68.9 |

| | | | | | | | | | | | |
|---|---|---|---|---|---|---|---|---|---|---|---|
| *Halorubrum persicum* C49[T] | NHOA00000000 | 3,578,349 | 158 | 56,313 | 98.94 | 0.51 | 3,567 | 3,513 | 3 | 49 | 65.9 |
| *Halorubrum saccharovorum* DSM 1137[T] | AOJE00000000 | 3,423,703 | 72 | 183,455 | 99.25 | 0 | 3,304 | 3,251 | 3 | 48 | 66.9 |
| *Halorubrum sodomense* RD 26[T] | FOYN00000000 | 3,030,553 | 9 | 917,372 | 99.19 | 0.51 | 3,053 | 2,999 | 5 | 47 | 69.0 |
| *Halorubrum tebenquichense* DSM 14210[T] | AOJD00000000 | 3,328,860 | 93 | 75,545 | 99.68 | 0.76 | 3,297 | 3,242 | 4 | 49 | 68.0 |
| *Halorubrum terrestre* JCM 10247[T] | AOIW00000000 | 3,376,225 | 79 | 97,508 | 99.49 | 0 | 3,370 | 3,312 | 10 | 46 | 68.0 |
| *Halorubrum trapanicum* CBA1232 | AP017569 | 2,835,595 | 1 | 2,835,595 | 99.38 | 0.51 | 2,982 | 2,926 | 6 | 48 | 69.6 |
| *Halorubrum vacuolatum* DSM 8800[T] | FZNQ00000000 | 3,477,860 | 72 | 153,076 | 98.92 | 0.84 | 3,373 | 3,307 | 5 | 59 | 62.9 |
| *Halorubrum xinjiangense* CGMCC 1.3527[T] | FNBO00000000 | 3,261,379 | 25 | 212,408 | 99.57 | 0.38 | 3,203 | 3,142 | 9 | 50 | 68.3 |

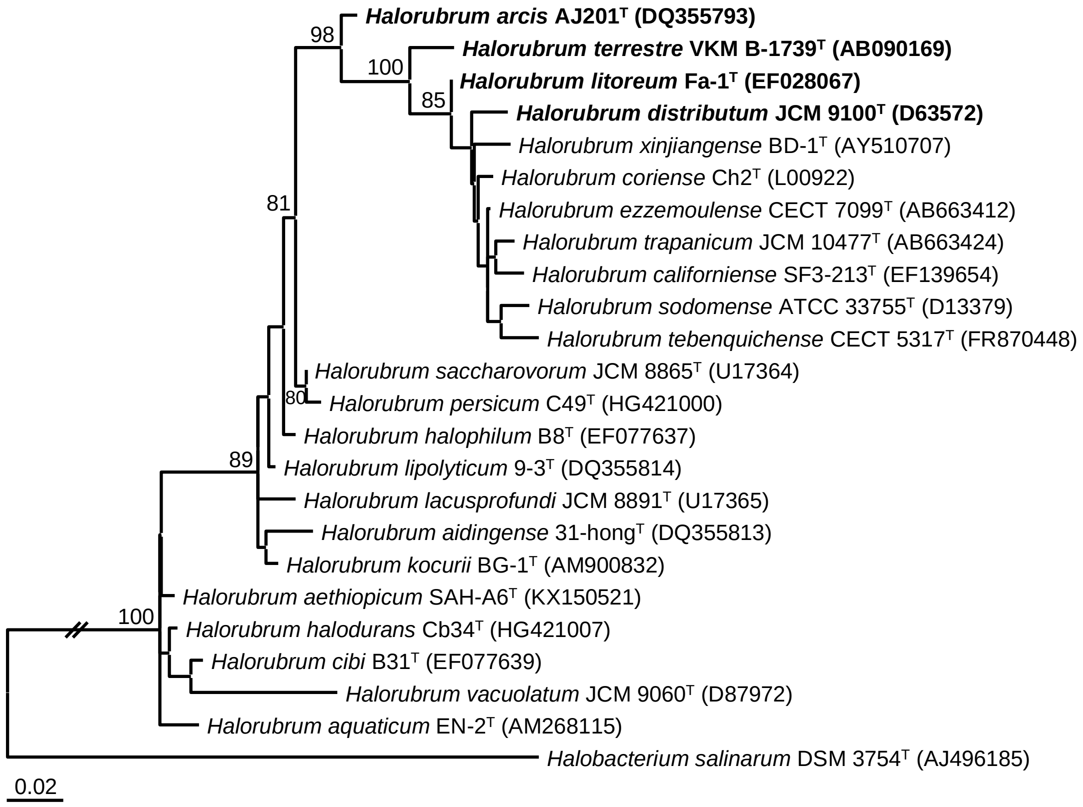


**Supplementary Fig. S1.** Maximum likelihood phylogenetic tree based on 16S rRNA gene sequences comparison showing the relationship between *Halorubrum distributum*, *Halorubrum terrestre*, *Halorubrum arcis* and *Halorrubrum litoreum* and other related species of the genus *Halorubrum*. The accession numbers of the sequences used are shown in parentheses after the strain designation. Bootstrap values (%) based on 1000 replicates are shown for branches with more than 70 % bootstrap support. *Halobacterium salinarum* DSM 3754$^{T}$ was used as outgroup. The scale bar represents 0.02 substitutions per nucleotide position.

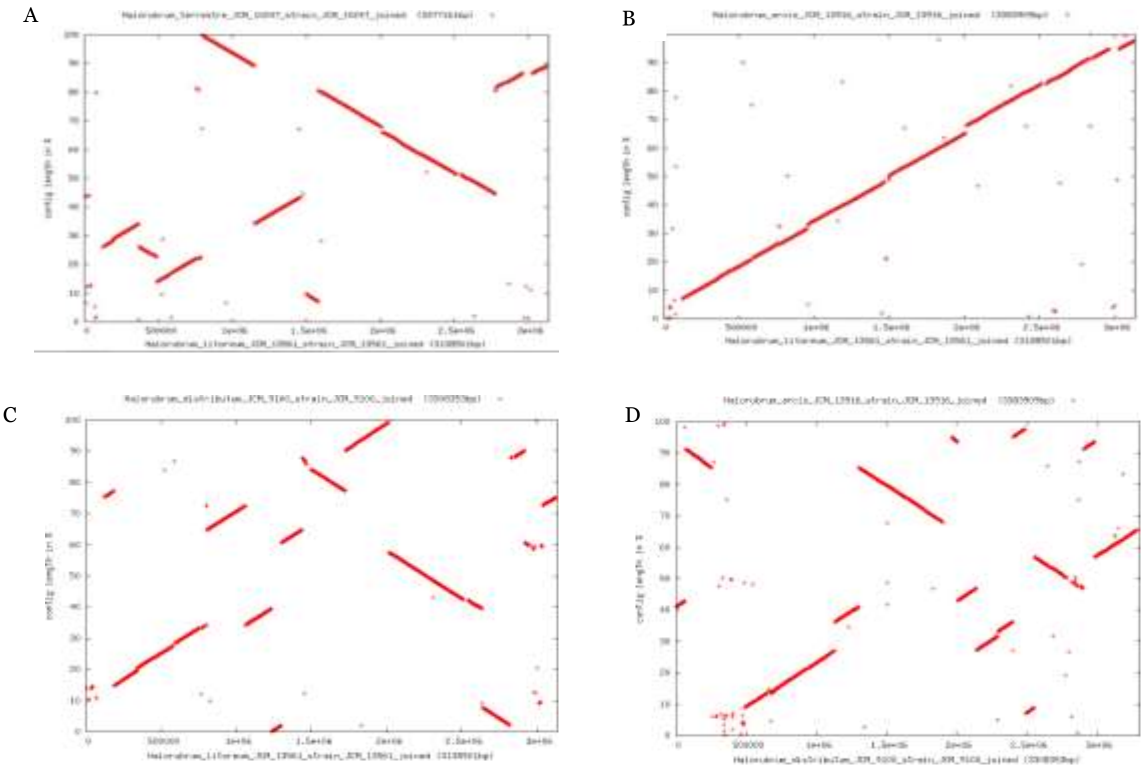

**Supplementary Figure S2.** Synteny plots between the proposed synonymous species. A, *Halorubrum litoreum* JCM 13561$^T$ vs *Halorubrum terrestre* JCM 10247$^T$; B, *Halorubrum litoreum* JCM 13561$^T$ vs *Halorubrum arcis* JCM 13916$^T$; C, *Halorubrum litoreum* JCM 13561$^T$ vs *Halorubrum distributum* JCM 9100$^T$*;* D, *Halorubrum distributum* JCM 9100$^T$ vs *Halorubrum arcis* JCM 13916$^T$.

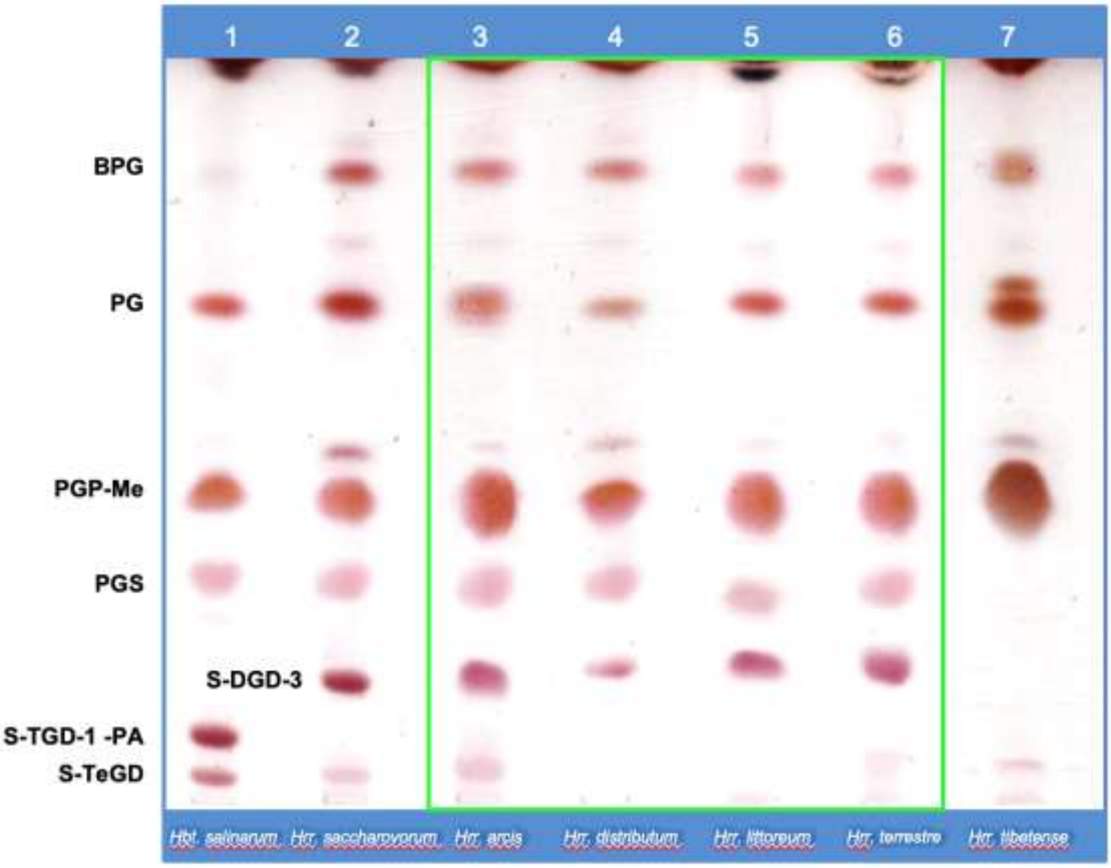


**Supplementary Figure S3.** One-dimensional High Performance Thin Layer Chromatography (HPTLC) of polar lipids extracted from *Halorubrum* strains in this study and other haloarchaeal reference species. The plate was revealed with sulfuric acid 5 % in water and charred by heating at 160 °C.

**Lanes:** 1, *Halobacterium salinarum* DSM 3754$^{T}$; 2, *Halorubrum saccharovorum* DSM 1137$^{T}$; 3, *Halorubrum arcis* JCM 13916$^{T}$; 4, *Halorubrum distributum* JCM 9100$^{T}$; 5, *Halorubrum litoreum* JCM 13561$^{T}$; 6, *Halorubrum terrestre* JCM 10247$^{T}$; 7, *Halorubrum tibetense* JCM 11889$^{T}$.

**Abbreviations:** BPG, biphosphatidylglycerol; PG, phosphatidylglycerol; PGP-Me, phosphatidylglycerol phosphate methyl ester; PGS, phosphatidylglycerol sulfate; S-DGD-3, sulfated mannosyl glucosyl diether; S-TGD-1-PA, glycocardiolipin (sulfated triglycosyl diphytanyl archaeol ester linked to phosphatidic acid); S-TeGD, sulphated tetraglycosyl diether.
